# Intraspecific Genetic Diversity of *Cistus creticus* L. and Evolutionary Relationships to *Cistus albidus* L. (Cistaceae): Meeting of the Generations?

**DOI:** 10.3390/plants10081619

**Published:** 2021-08-06

**Authors:** Brigitte Lukas, Dijana Jovanovic, Corinna Schmiderer, Stefanos Kostas, Angelos Kanellis, José Gómez Navarro, Zehra Aytaç, Ali Koç, Emel Sözen, Johannes Novak

**Affiliations:** 1 Institute of Animal Nutrition and Functional Plant Compounds, University of Veterinary Medicine Vienna, Veterinaerplatz 1, 1210 Vienna, Austria; Dijana.Jovanovic@vetmeduni.ac.at (D.J.); corinna.schmiderer@boku.ac.at (C.S.); Johannes.Novak@vetmeduni.ac.at (J.N.); 2 Department of Horticulture, School of Agriculture, Aristotle University of Thessaloniki, 541 24 Thessaloniki, Greece; skostas@agro.auth.gr; 3 Group of Biotechnology of Pharmaceutical Plants, Laboratory of Pharmacognosy, Department of Pharmaceutical Sciences, Aristotle University of Thessaloniki, 541 24 Thessaloniki, Greece; kanellis@pharm.auth.gr; 4 Botanical Institute, Systematics, Ethnobiology and Education Section, Botanical Garden of Castilla-La Mancha, Avenida de La Mancha s/n, 02006 Albacete, Spain; jgon0141@yahoo.es; 5 Department of Field Crops, Faculty of Agriculture, Eskişehir Osmangazi University, Eskişehir 26480, Turkey; zehrak@ogu.edu.tr (Z.A.); alikoc@ogu.edu.tr (A.K.); 6 Department of Biology, Botany Division, Science Faculty, Eskişehir Technical University, Eskişehir 26470, Turkey; esozen@eskisehir.edu.tr

**Keywords:** *Cistus*, *Cistus creticus*, genetic diversity, ITS, Mediterranean, phylogeography, *rpl32-trnL*, *trnL-trnF*

## Abstract

*Cistus* (Cistaceae) comprises a number of white- and purple-flowering shrub species widely distributed in the Mediterranean basin. Within genus *Cistus,* many taxa are subject to various taxonomic uncertainties. *Cistus creticus*, a prominent member of the purple-flowered clade, is a prime case of the current taxonomic troubles. Floras and databases approve different species names and utilise different or additional/fewer synonyms. Various intraspecific classification systems based on subspecies or varieties are in use. The inconsistent determination of plant material makes it difficult to compare literature regarding the phytochemical diversity and biological activities of plant material and impedes a systematic utilization of the manifold medicinal properties of *C. creticus*. In the present investigation, we used DNA sequence data from one nuclear region (ITS) and two chloroplast regions (*trnL-trnF*, *rpl32-trnL*) to test the intraspecific genetic diversity of *C. creticus* and its evolutionary relationships to the closely related *C. albidus*. The combined DNA data confirmed *C. creticus* as a rather heterogeneous species that integrates two major evolutionary lineages with clearly different genetic characteristics. The ‘Eastern Mediterranean clade’ seems to represent old and ancestral characteristics. This lineage exhibits a close relationship to the geographically distant *C. albidus,* expressed by very closely related ribotypes and an interspecifically shared chlorotype. The ‘Western Mediterranean clade’ is characterized by a distinctive ITS polymorphism (co-occurring paralogous ribotypes) and more distantly related chlorotypes. The formation of the genetically complex ‘Western Mediterranean clade’ seems to have involved hybridization and recurrent formation or migration movements.

## 1. Introduction

The genus *Cistus* L. (Cistaceae, Malvales) comprises white- and purple-flowering shrub species that are widely distributed in the Mediterranean, on the Canary Islands, and on Madeira, often in open or disturbed, fully sun-exposed vegetation. Various monographs on *Cistus* (e.g., [1,2,3,4,5]) have recognized between 16 and 28 species and proposed conflicting intrageneric classifications. In the Euro+Med PlantBase, 31 *Cistus* species were listed [6]. The latest taxonomic treatment of the 14 native Iberian species [7] recognized three subgenera, *Cistus* L. (including the purple-flowering species), *Leucocistus* Willk. and *Halimioides* (Willk.) Demoly & P. Monts (both including white- and whitish-pink-flowering species). Molecular phylogenetic analysis based on various chloroplast markers and nuclear ITS and ncp*GS* have in the main supported the proposed intrageneric organization [8,9,10,11].

Within the genus *Cistus*, there exist many uncertainties concerning taxonomic entities at lower systematic levels. Hybridization and a high degree of morphological polymorphism complicate the determination of species boundaries. Local Mediterranean floras refer to different classification systems resulting in the inconsistent naming of taxa and a plethora of synonyms and specific epithets difficult to interpret. This is all true for *C. creticus* L., a prime case of the current taxonomic troubles. *Cistus creticus* is a member of the purple-flowered clade and one of the few *Cistus* species widely distributed in the Eastern Mediterranean. The species name *C. creticus* seems to be widely accepted but the synonyms *C. incanus* auct. and *C. villosus* L. have been also frequently applied in Floras and in recent literature. The Euro + Med PlantBase [6] acknowledges *C. creticus* as the accepted species name and mentions two homotypic synonyms (*C. incanus* subsp. *creticus* (L.) Heywood and *C. villosus* subsp. *creticus* (L.) Nyman) and two heterotypic synonyms (*C. garganicus* Ten. and *C. polymorphus* Willk., nom. illeg.). Other established plant databases (e.g., The Med-Checklist, The Plant List, ITIS, TROPICOS, Mansfeld’s World Database of Agriculture and Horticultural Crops) approve a different species name, give different or fewer/additional synonyms, or acknowledge synonyms recognized elsewhere as accepted species names on a par.

According to phylogenetic studies [8,9,10,11], *C. creticus* appears to be a good taxonomic entity. However, only few (and mostly Western Mediterranean) accessions were investigated in each case and *C. creticus* appears to be highly variable with some geographical structuring. Based on morphological and phytochemical characteristics, some authors have recognized three subspecies, *C. creticus* subsp. *eriocephalus* (Viv.) Greuter & Burdet, *C. creticus* subsp. *corsicus* (Loisel.) Greuter & Burdet, and *C. creticus* L. subsp. *creticus* [12,13,14,15]. Ref. [15] demonstrated that Corsican and Sardinian subsp. *eriocephalus* and subsp. *corsicus* could be differentiated by ISSR data as well as by morphological (absence/presence of glandular trichomes) and phytochemical characteristics. [16] detected a correlation between chloroplast haplotype differentiation and ecological factors (e.g., geology). However, the current knowledge of intraspecific genetic diversity of *C. creticus* is restricted to these island populations from the western range of the species’ distribution and it is difficult to assess how the morphological and phytochemical variability is correlated with genetic differentiation. The poor knowledge of intraspecific genetic diversity of *C. creticus* also makes it difficult to assess the relationship to the genetically closely related *C. albidus* L.

Within the purple-flowered *Cistus* clade, four species, namely *C. creticus*, *C. albidus*, *C. heterophyllus* Desf., and *C. crispus* L. consistently form a well-supported, paraphyletic group [8,9,10,11]. Within this group, *C. creticus* and *C. albidus* appear to be closely related but their specific evolutionary relationships remain unresolved. Based on comprehensive sequence and pollen analysis, Ref. [11] suspected *C. creticus* and *C. albidus* to be either two sub-units of a large ancestral species that fragmented into two geographical entities with limited outcrossing barrier in between or, alternatively, to be two ancient species now differentiated enough to remain distinct even when in sympatry. Both species exhibited rather similar or identical sequence characteristics at some chloroplast and nuclear loci. Apart from their close genetic relationship, however, they can be easily distinguished by discrete morphological characteristics as e.g., indumentum and leaf size and shape [7,17]. The distribution area of *C. creticus* and *C. albidus* overlaps very little. *Cistus creticus* is native to parts of North Africa, absent in Portugal, Spain (with the one exception described below) and France and then again present in the Central and Eastern Mediterranean, from Italy to the Near East. *Cistus albidus* is exclusively present in the Western Mediterranean and therewith mainly in the geographical area were *C. creticus* is rare or absent (North Africa to Portugal, Spain, France and Western Italy). In Morocco and along the Italian Ligurian coast, the distribution of both species might overlap [11]. Sympatric occurrence of *C. creticus* and *C. albidus* was also described for a restricted area of South-Spain were some small relict populations of *C. creticus* and few natural fertile hybrids with *C. albidus* (*C*. × *canescens* Sweet) were discovered [17,18].

The resin of *C. creticus* is rich in labdane-type diterpenes that are of scientific and medicinal interest as they show significant cytotoxic activities against e.g., human leukemic and breast cancer cell lines [19,20,21,22,23]. Further valuable secondary compounds present in *C. creticus* are diverse polyphenolic compounds (e.g., gallic acid and flavonoids such as myricitrin and quercitrin [22,24,25]. Plant material of *C. creticus* could be an important natural source for these secondary compounds but the confusing taxonomic circumstances result in trade difficulties with synonymously specified plant material and inconsistent drug qualities (Paula Torres Londoño, personal communication). The inconsistent determination of plant material also makes it difficult to compare literature on volatile characteristics of *Cistus* accessions or biological activity of *Cistus* preparations.

The aim of this investigation was to provide a sound basis for the ongoing discussion about species names, species concept and (practical) relevance of taxonomic sub-entities. We used chloroplast (*trnL-trnF*, *rpl32-trnL*) and nuclear (ITS) DNA sequence data for a comprehensive evaluation of *C. creticus.* The specific aims of this investigation were: (1) to assess whether the various scientific names that have been used synonymously all refer to the same species that can be clearly distinguished from all other *Cistus* species, (2) to investigate whether the striking intraspecific variability is more due to continuous variation over the species distribution area or to the presence of distinct subspecies, and (3) to discuss the evolutionary relationships to the closely related *C. albidus.*

## 2. Material and Methods

### 2.1. Plant Material

Individual plants of *C. creticus* L., *C. albidus* L., and *C.* × *canescens* Sweet (*C. creticus* × *C. albidus*) were sampled during excursions to Albania, Croatia, Cyprus, France, Italy, Portugal, Spain and Turkey, in autumn 2016 and late spring 2017 and 2018. The plant material from the Greek *C. creticus* plants was collected by the AUTH in December 2018 (Ministry of Environment and Energy/Protection of Forests: 175958/5915, 22 November 2018). Additionally, seeds of *C. creticus* and *C. albidus* were obtained from the Millennium Seedbank (Royal Botanic Gardens Kew) and the Seed Bank Berlin Dahlem. Populations from these seeds were grown in the greenhouse of the University of Veterinary Medicine Vienna, Austria. Geographical coordinates of the native populations sampled as well as seedbank accession numbers are summarized in Supplementary Table S1. Two individual plants of each population were sequenced, with exception of the Cypriot populations (where the twelve analysed plants represent five geographically determined population clusters) and one Greek population where only one single sample was available. In summary, 67 populations (125 individual plants) of *C. creticus*, 15 populations (30 individual plants) of *C. albidus* and one hybrid population of *Cistus* × *canescens* (two individual plants) were analysed. Individual plants of *C. crispus* L. (two), *C. ladanifer* L. (two), *C. monspeliensis* L. (six), *C. parviflorus* L. (four), and *C. salviifolius* L. (thirteen) were co-sampled and included for comparative purposes in the whole analysis procedure. All plant material of wild populations was sampled in accordance with the guidelines of the Nagoya protocol (https://www.cbd.int/abs/text, accessed on 31 May 2018). Species were identified by following the keys of the local floras (Supplementary Table S1). Pictures of selected plants and populations are available as supplementary material in [25]. Voucher specimens are kept at the herbarium of the Institute for Animal Nutrition and Functional Plant Compounds, University of Veterinary Medicine Vienna, Austria.

### 2.2. DNA Extraction, Amplification and Sequencing

Genomic DNA was extracted from young, dried leaves using a CTAB extraction protocol based on [26]. Modifications are described in [27] (CTAB-protocol I). Genomic DNA of Greek genotypes was extracted from approximately 0.1 g of fresh leaves using a NucleoSpin Plant ΙΙ kit (Macherey Nagel, Schkeuditz, Germany) according to the manufacturer’s instructions. The nuclear ITS region was amplified using primers ITS5 and ITS4 ([28], modified by [29]). For amplification of the two plastid regions the primers e and f (*trnL-trnF* intergenic spacer, [30]) and *rpl32-trnL*-F and *rpl32-trnL*-R (*rpl32-trnL* intergenic spacer, [31]) were used. For a 15 µL PCR reaction, 1 µL of genomic DNA (5-10 ng) was added to a master mix containing 1x PCR buffer B, 2.5 mM MgCl_2_, 133 µM dNTP mix, 0.6 units Taq HOT FIREPol^®^ DNA Polymerase (all reagents Solis BioDyne, Tartu, Estonia), and 0.6 µM forward and reverse primers (Life Technologies, Vienna, Austria).

The PCR cycle profile for the amplification of nrITS included a denaturation step at 95 °C for 15 min, followed by 35 cycles of 95/58/72 °C (depending on the samples) for 45/45/60 s and a final elongation step of 7 min at 72 °C. Amplification of the *trnL-trnF* intergenic spacer started with an initial cycle of 95/45/72 °C for 15/1/1 min, followed by 35 cycles of 95/50/72 °C for 1/1/1 min and a final elongation step at 72 °C for 7 min. The cycle profile for the amplification of the *rpl32-trnL* intergenic spacer started with an initial cycle of 95/56/72 °C for 15 min/45 s/60 s, followed by 35 cycles of 95/56/72 °C for 30/60/120 s and a final elongation step with 72 °C for 10 min.

PCR products were checked on 1.4% agarose gels and purified with ExoI and FastAP (Fisher scientific, Vienna, Austria) according to the manufacturer‘s instructions. Sequencing was performed by an external company (Microsynth, Vienna, Austria) using the primers ITS5, f or *rpl32-trnL*-F as sequencing primers.

The obtained sequences were edited using Geneious 5.3.6 (www.geneious.com). Concerning nrITS, some directly sequenced accessions of *C. creticus* (about 27%) and *C. albidus* (about 1%) resulted in ambiguous sequence chromatograms characterized by the presence of single to few additive polymorphic sites and noise/divergence of the sequences after a specific alignment position. These sequence chromatogram characteristics could later be attributed to an intraspecific/intraindividual ITS sequence polymorphism, namely the simultaneous occurrence of divergent ITS paralogs differing by one to five SNPs and one indel of 6 bp in the last third of the sequences. For individuals exhibiting different paralogs in somehow equivalent copy numbers (additive polymorphic sites of the same height) the two different ribotypes were determined through careful base subtraction (based on [32]), by using clear sequence chromatograms of geographically neighboured accessions as reference sequences. In some plants solely the predominant ribotype was extracted. All ITS sequences deriving from heterogeneous individuals were labelled as such and were handled with special attention in the subsequent data analysis. Within the individuals of white-flowering *Cistus* species no sequence characteristics or sequencing artefacts were detected that would indicate a regular simultaneous presence of ITS variants. All sequences were deposited in GenBank (accession numbers are provided in Supplementary Table S1). For accessions with intra-individual ITS polymorphism, two accession numbers per plant individual were assigned.

### 2.3. Sequence Analysis and Phylogenetic Reconstruction

ITS, *trnL-trnF*, and *rpl32-trnL* sequences were aligned using Geneious 5.3.6, with subsequent manual correction. Variable positions in the data matrices were checked against the original sequence chromatogram files. NrITS ribotypes and cpDNA chlorotypes were defined according to obvious patterns in the condensed alignments and sequences were grouped and assigned accordingly. The distribution of nrITS-ribotypes, *trnL-trnF* chlorotypes, and *rpl32-trnL* chlorotypes was then mapped to visualize phylogeographical distribution patterns among populations. Distribution maps were compiled with Google Earth (https://www.google.com/earth/download/), with subsequent modifications.

GenBank was searched for additional available sequences of purple-flowered *Cistus* species (*trnL-trnF*: EU684549-EU684567 [16], JF900439-JF900444 (Pawluczyk et al., 2012) [33], DQ093021, DQ093022, DQ093025-DQ093036, DQ093060, DQ093061 [8], FJ492017 [9], KY651347, KY651348 [34]; *rpl32-trnL*: EU684568-EU684586 [16], ITS: DQ092932, DQ092933, DQ092936-DQ092944, DQ092967 [8], KY651255, KY651256 [34], GQ281663, GQ281664 [10]). These GenBank accessions were included into the respective alignments and the subsequent analysis, with the main aim to demonstrate chloro-/ribotype relationships to and among the most closely related *Cistus* species. R 3.5.2 [35] and package pegas (version 0.14, default settings [36]) were used to visualize cpDNA chlorotype relationships. As significantly fewer *rpl32-trnL* IGS sequences of target and especially outgroup species were available, we refrained from combining the two chloroplast regions for haplotype analysis. Genealogical relationships among ITS ribotypes were assessed by a minimum spanning network constructed and visualized using R 3.5.2 [35] and the packages adegenet [37,38], poppr 2.8.1 [39,40] and magrittr [41]. The underlying distance matrix was calculated based on Provesti’s genetic distance.

## 3. Results

### 3.1. cpDNA Variability

**Figure 1:**
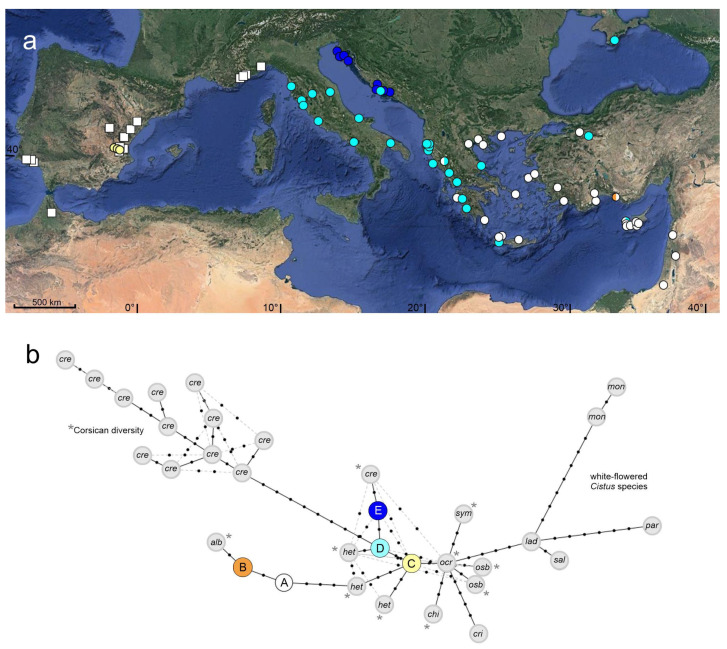
(**a**) Geographical distribution of *trnL-trnF* chlorotypes detected during this investigation. Squares represent populations of *C. albidus*, circles populations of *C. creticus* (two individual plants each), colours point to the different chlorotypes (chlorotype identification is designated in Figure 1b). (**b**) *trnL-trnF* chlorotype network constructed from the extended sequence alignment (Table S2, Supplementary Materials). Coloured circles represent *C. creticus* and *C. albidus* chlorotypes detected during this investigation. Grey circles indicate chlorotypes from additional sequences of the enlarged alignment [GenBank sequence data (*) and the sequences of *C. crispus* and white-flowered *Cistus* species created by authors]. Light grey lines indicate alternative relationships. alb = *C. albidus*, cre = *C. creticus*, chi = *C. chinamadensis*, cri = *C. crispus*, het = *C. heterophyllus*, lad = *C. ladanifer*, mon = *C. monspeliensis*, ocr = *C. ocreatus*, osb = *C. osbeckiifolius*, par = *C. parviflorus*, sal = *C. salviifolius*, sym = *C. symphytifolius*.

The *trnL-trnF* alignment representing the two target species *C. creticus* and *C. albidus* and their hybrid *C*. × *canescens* consisted of 157 sequences (125/30/2) and 427 bp. Eight nucleotide substitutions and three indels (two small indels related to poly-T stretches, one indel of four bp) were detected. When neglecting the poly-T stretch related indels, the remaining polymorphic sites identified five different *trnL-trnF* chlorotypes (variable *trnL-trnF* alignment positions are summarized in Table 1; geographical chlorotype distribution is displayed in Figure 1a). The frequent chlorotype A was shared by *C. creticus* and *C. albidus* (Figure 1a). Within *C. albidus* it was the only *trnL-trnF* chlorotype present in the 15 populations. Within *C. creticus* chlorotype A was not present in populations close to the natural distribution of *C. albidus* but was the dominant one in geographically distant Eastern Mediterranean areas (Greece, Turkey, Cyprus, and the Near East). Chlorotype B (a rare variant of chlorotype A) was detected in one Turkish population. The three *trnL-trnF* chlorotypes C, D and E were specific for *C. creticus*. Chlorotype C was present in the three, geographically isolated Spanish populations. Chlorotype D was distributed in Italy, Albania, Western Greece, and the Crimean Peninsula and showed sporadic presence in Croatia, Turkey and Cyprus. Chlorotype E was the main chlorotype of the Croatian populations. Of the two Spanish accessions of the hybrid of *C. creticus* and *C. albidus* (*C.* × *canescens*), one exhibited chlorotype A, the other one chlorotype C.

For network analysis, additional sequences (GenBank sequences and sequences created by authors) of *C. creticus* (21), *C. albidus* (6), purple-flowered *C. chinamadensis* (2), *C. crispus* (5), *C. heterophyllus* (5), *C. ocreatus* (2), *C. osbeckiifolius* (3), *C. symphytifolius* (2) and white-flowered *C. ladanifer* (2), *C. monspeliensis* (6), *C. parviflorus* (appeared most closely related to white-flowered species although it possesses light purple flowers, [8]) (4) and *C. salviifolius* (13) were included. The therewith enlarged alignment consisted of 228 sequences and 480 bp. Forty-six variable nucleotide positions and 14 indels identified 33 *trnL-trnF* chlorotypes (Figure 1b; Supplementary Table S2). The *trnL*-*trnF* haplotype network (Figure 1b) depicted chlorotype A (*C. albidus* and Eastern Mediterranean *C. creticus*) and the closely related, rare chlorotype B distinct and isolated from the further chlorotypes of *C. creticus*. Chlorotype A was connected via five mutations (plus one poly-T stretch related indel) and one chlorotype of *C. heterophyllus* to chlorotype C, the chlorotype of the three Spanish relict populations of *C. creticus*. During this investigation, chlorotype C was solely detected in Spanish *C. creticus* but three GenBank accessions indicated that chlorotype C might be interspecifically shared with *C. albidus* and *C. heterophyllus*. The relationship of chlorotypes C, D, and E with the three further *C. heterophyllus* chlorotypes appeared to be close and alternative relationships between these chlorotypes and to chlorotypes of *C. ocreatus* and *C. osbeckiifolius* were indicated. Chlorotype C of the Spanish populations was (via *C. ocreatus*) connected to the chlorotypes of five other purple-flowered *Cistus* species (except for *C. crispus*, all endemic to the Canary Islands). Via one substitution (plus one poly-T stretch related indel and one 4 bp indel), chlorotype C was linked to chlorotype D, the immediate ancestor of chlorotype E (one mutation plus one poly-T stretch related indel in two accessions) and of the very distinct impressive haplotype diversity of Corsica and Sardinia, where 19 accessions exhibited twelve chlorotypes (characterized by combinations of 18 unique nucleotide polymorphisms; Supplementary Table S2). The white-flowered clade was connected via *C. ocreatus* and *C. ladanifer*.

**Figure 2:**
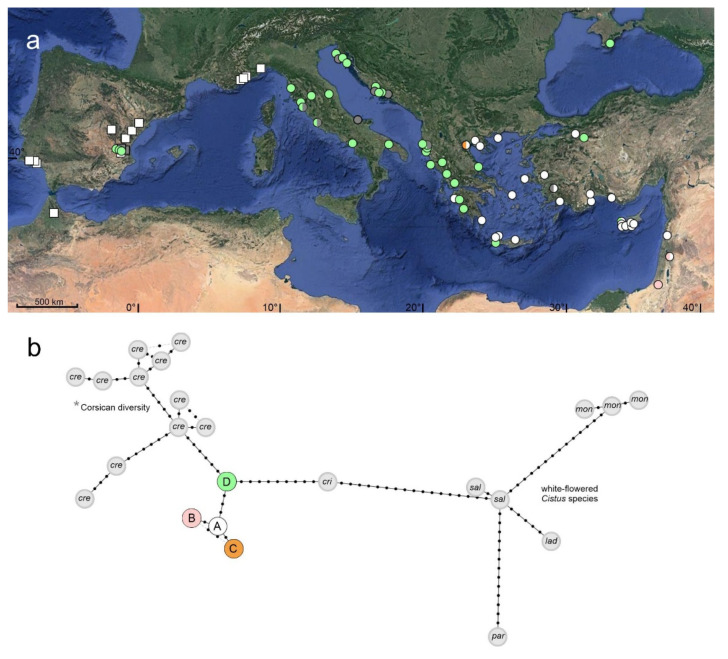
(**a**) Geographical distribution of *rpl32-trnL* chlorotypes detected during this investigation. Squares represent populations of *C. albidus*, circles populations of *C. creticus* (two individual plants each). Colours point to the different chlorotypes (chlorotype identification is designated in Figure 2b) or indicate missing data (dark grey). (**b**) *rpl32-trnL* chlorotype network constructed from the extended sequence alignment (Table S3, Supplementary Materials). Coloured circles represent *C. creticus* and *C. albidus* chlorotypes detected during this investigation. Grey circles indicate chlorotypes from additional sequences of the enlarged alignment [GenBank sequence data (*) and the sequences of *C. crispus* and white-flowered *Cistus* species created by authors]. Light grey lines indicate alternative relationships. cre = *C. creticus*, cri = *C. crispus*, lad = *C. ladanifer*, mon = *C. monspeliensis*, par = *C. parviflorus*, sal = *C. salviifolius*.

The *rpl32-trnL* alignment representing *C. creticus*, *C. albidus* and *C*. × *canescens* collected during this investigation consisted of 141 sequences (113/26/2) and 899 bp. Five nucleotide substitutions identified four *rpl32-trnL* chlorotypes (Table 2, Figure 2a). Chlorotype A was shared by *C. creticus* and *C. albidus* (Table 2, Figure 2a) and was detected in most plants exhibiting *trnL-trnF* chlorotype A (Figure 1a and Figure 2a). Local sequence variants of the *rpl32-trnL* chlorotype A were present in two Israeli and one Jordanian accession (chlorotype B) as well as one Greek accession (chlorotype C). Chlorotype D was specific to *C. creticus* and was present in Spain, Italy, Croatia, Albania, Western Greece, the Crimean Peninsula and in one Cypriot and one Turkish population (plants exhibiting *trnL-trnF* chlorotypes C, D and E).

When including additional sequences (GenBank sequences and sequences created by authors) of *C. creticus* (19), purple-flowered *C. crispus* (2) and white-flowered *C. ladanifer* (2), *C. monspeliensis* (6), *C. parviflorus* (4), and *C. salviifolius* (13), the *rpl32-trnL* alignment consisted of 188 sequences and 916 bp. Seventy-one nucleotide substitutions and eight indels identified 23 *rpl32-trnL* chlorotypes (Figure 2b; Supplementary Table S3). In the *rpl32-trnL* haplotype network (Figure 2b) chlorotype A (immediate ancestor of the local variants B and C) is connected via three substitutions to chlorotype D. Chlorotype D functions as a link to the third included species of the purple-flowered clade, *C. crispus* and was identified as ancestor of the remarkable Corsican/Sardinian haplotype diversity (14 haplotypes from 19 individual plants, defined by 21 substitutions specific for the island populations). The white-flowered clade is linked via *C. salviifolius* and, in the absence of sequences from further, purple-flowered species, *C. crispus*.

### 3.2. nrITS Variability

One hundred and fifty-seven individual plants of *C. creticus*, *C. albidus* and *C*. × *canescens* (125/30/2) were directly sequenced for ITS. From 32 (22/8/2) accessions no usable ITS sequence could be obtained, in most cases due to competing fungal (e.g., *Aureobasidium* sp.) DNA. Among them were also the sequences of the two available hybrid individuals and no conclusion about ITS sequence additivity in putative F1 individuals could be drawn. The bulk of the 125 successfully sequenced accessions exhibited clear sequence chromatograms and was uncomplicated to edit. Twenty-eight direct sequences of *C. creticus* (25 accessions, 27% of the successfully sequence plants) and *C. albidus* (3 accessions, 1% of the successfully sequence plants), however, showed very particular characteristics, single additive polymorphic sites in ITS1 and ITS2 and a short series of ambiguous bases close to the ending of ITS2. A comparison of such ambiguous sequence chromatograms with unambiguous ones suggested the presence of intragenomic ITS variants of different lengths. The stable patterns of additive polymorphic sites and shifted nucleotides could usually be fully explained by the simultaneous occurrence of two ITS sequence variants: one ITS variant continuously present in the whole sampled area and one of solely regionally occurring ITS ribotypes. For those heterozygous plants exhibiting two different ribotypes in equivalent copy numbers, the two ITS variants were extracted by careful base subtraction and using sequences from geographically close homozygous individuals as references. For some heterozygous plants, where one ITS variant was underrepresented, the dominant ITS ribotype was extracted. Such extracted sequences of heterozygous plants were labelled and handled with special care in the subsequent analysis and result interpretation.

The ITS alignment representing the accessions of the target species *C. creticus*, *C. albidus* and *C*. × *canescens* collected during this investigation consisted of 143 sequences (118/25/0, with 38/6/0 of them derived from heterozygous plants) and 699 bp. Twenty-nine ITS ribotypes were defined based on 25 variable nucleotide positions and a 6 bp indel. One hundred and nine ITS sequences (51/13/18/27) were ascribed to one of the four main ribotypes A, B, C or D (Table 3). These four main ribotypes, each, are well backed by the high number of clear direct ITS sequence chromatograms. Thirty ITS sequences (including eight sequences that were isolated solely from heterozygous plants) exhibited the sequence characteristics, defining one main ribotype in combination with few additional individual SNPs and/or additive polymorphic sites that often seem to depict a transition between two main ribotypes. Five ITS variants from heterozygous plants exhibited individual SNPs (ribotypes E/F).

**Figure 3:**
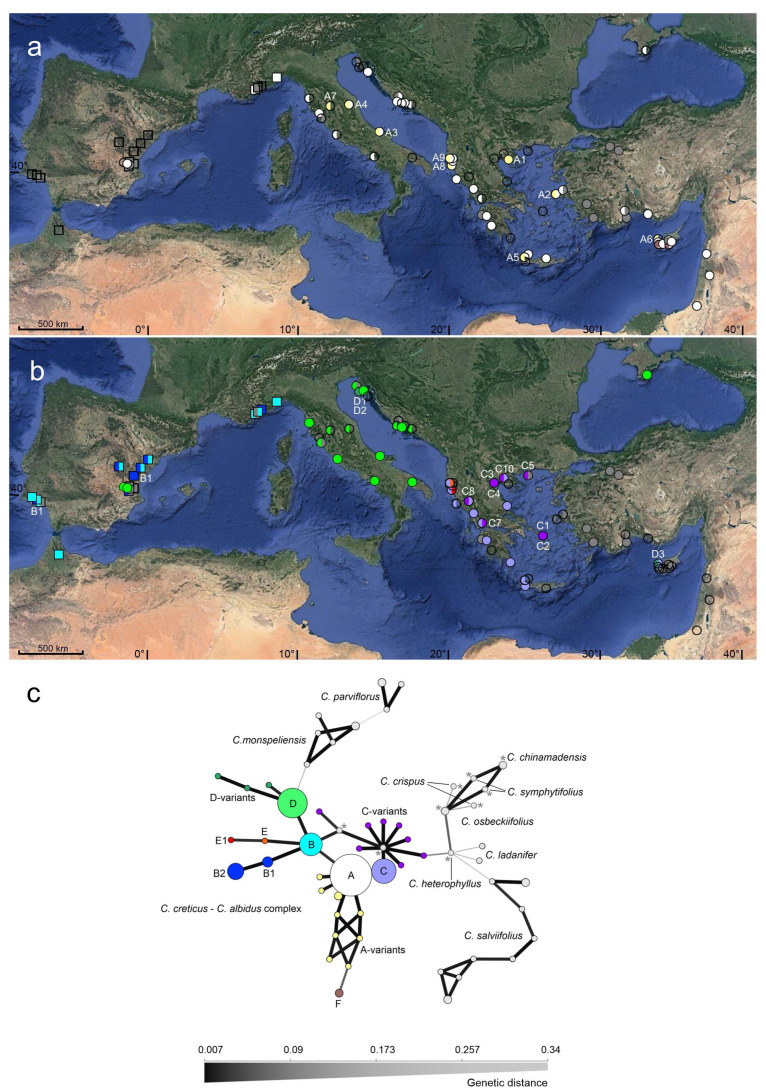
Geographical distribution of ribotypes A and F (and rare variants; (**a**)) and ribotypes B, C, D and E (and their rare variants; (**b**)). For a clearer presentation, the overlapping distribution of ribotype A and ribotypes B, C, D and E was illustrated on two separate figures. Squares represent populations of *C. albidus*, circles populations of *C. creticus* (usually two individual plants each). Colours of squares and circles point to the six main ribotypes (and their variants) or indicate missing data (dark grey). Rare variants were identified according to the numbering in Table 3. (**c**) Minimum spanning network based on the extended ITS sequence alignment (Prevosti’s absolute genetic distance). Coloured circles represent the ribotypes of the *C. creticus*–*C. albidus* complex detected during this investigation (circle colours reflect those used in Figure 3a,b to display ribotype distribution). Grey circles represent ribotypes of different *Cistus* species or ribotypes based on GenBank sequence information (labelled with *). Circle areas reflect the sample sizes. Edge widths and shading represent relatedness.

Within *C. creticus* ribotype A was frequent and continuously present in the whole sampled area, with by trend stronger or even exclusive appearance in populations of the Eastern Mediterranean area. Nine local variants of ribotype A (A1–A9, Figure 3b), differing by one or two additional nucleotide substitutions or single additive polymorphic sites, were present in Italy, Albania, Greece, and Cyprus. Within *C. albidus*, ribotype A was present in three heterozygous plants of France and Italy, at the eastern margins of the species distribution. The regular ITS variant of *C. albidus* was ribotype B*,* inter-specific variants (B1/B2, Figure 3b) with one additional additive polymorphic site/nucleotide polymorphism were present from Portugal to France. Within *C. creticus*, the distribution of ribotype A overlapped with that of further species-specific ribotypes (C, D, E, F, and G; Figure 3b). Between Spain and Greece, *C. creticus* plants were either homozygous for ribotype A or homozygous for the regionally present co-occurring ribotype, or they were heterozygous and exhibited both, ribotype A and the regionally present second ribotype. Ribotype C was co-occurring in Albanian and Greek populations, ten rare local variants of ribotype C seem to constitute intermediate sequence variants between ribotype C and ribotype B and/or exhibited single additional mutations. Ribotype D was locally distributed in Italian and Croatian populations, and its three rare variants exhibited one additional individual mutation or seemed to constitute the intermediate sequence variant between ribotype D and ribotype B. The rare Albanian ribotype E (and its variant E1) seemed to constitute an early intermediate between ITS variant B and C that gained one additional mutation. Ribotype F, exclusively distributed in Cyprus, exhibited sequence characteristics that were not present in any other *C. creticus* ribotype.

When including additional sequences (GenBank sequences and sequences created by authors) of *C. creticus* (2), *C. albidus* (3), purple-flowered *C. chinamadensis* (2), *C. crispus* (2), *C. heterophyllus* (1), *C. osbeckiifolius* (2), *C. symphytifolius* (2), and white-flowered *C. ladanifer* (2), *C. monspeliensis* (6), *C. parviflorus* (4), and *C. salviifolius* (11), the final ITS alignment consisted of 180 sequences and 705 bp. A total of 57 distinct ITS sequence variants were defined based on nucleotide polymorphisms at 101 variable alignment positions and all-over 6 indels (Supplementary Table S4). A minimum spanning network (Figure 3c) was calculated to depict ribotype relationships. The widespread ribotype A (plus its local rare variants) was connected via ribotype B, the ribotype specific for *C. albidus* and the supposed common ancestor of the further main ribotypes of *C. creticus*, the Italian/Croatian lineage D (plus its variants), the Albanian/Greek lineage C (plus its variants) and the rare Albanian lineage E/E1. The distinct Cypriot ribotype F, although devoid of the characteristic indel, was linked to ribotype A. The five additional species of the purple-flowered clade were connected via the one ITS variant of *C. heterophyllus* and one rare ITS variant of *C. creticus* designated to ITS lineage C. The four white-flowering species were split up. *Cistus monspeliensis* and *C. parviflorus* were connected via *C. creticus* ribotype D. *Cistus ladanifer* and *C. salviifolius* were connected via *C. heterophyllus* that also linked the further, purple-flowered species.

## 4. Discussion

### 4.1. Intragenomic ITS Diversity in C. creticus

*Cistus albidus* appeared as a quite homogenous species, characterized by a single chlorotype and little ribotype variability. *Cistus creticus* was remarkably diverse, with distinct chloro- and ribotypes and clear evidence for intraindividual ITS polymorphism in about 27% of the successfully sequenced accessions. The presence of intragenomic ITS variants in *C. creticus* has not explicitly been described before but GenBank sequences ending after the position of the 6 bp indel (starting point of continuously shifted bases in the direct sequence chromatogram) could indicate that previous investigations included consensus sequences from heterozygous accessions. The ITS ribotypes described here were not verified by cloning and the rare ITS variants need confirmation. However, the four frequent ribotypes (A, B, C, D) are confirmed by a bulk of clear direct sequence chromatograms. For most heterozygous accessions of *C. creticus*, base ambiguities, and base shifts in their direct ITS sequence chromatograms could be fully elucidated by simultaneous occurrence of the continuously widespread ribotype A and one of the two further, regionally distributed main ribotypes (C and D). These stable patterns strongly suggest the presence of persistent paralogous ribosomal loci. It was hypothesized that the location of ribosomal loci on different chromosomes might have a preventative effect against homogenization through concerted evolution [42]. A detailed investigation of 45S rDNA site number and location in *Cistaceae*, however, provided no evidence for the presence of more than one ribosomal locus within *C. creticus* [43]. Regarding the purple-flowered *Cistus* clade, the results of [43] indicated that, except for the basal *C. crispus*, all purple-flowered species diversified from a common ancestral lineage with one single rDNA site. For their investigation [13] analysed two *C. creticus* accessions that were also present in our study (SC19 and SC23). Both populations appeared heterogeneous, with one plant that resulted in a clear ITS sequence chromatogram and a second one that showed clear evidence for intragenomic ITS variation. Probably, rDNA site number in *C. creticus* was underestimated when [13] picked two homogenous accessions by chance.

### 4.2. Geographical Distribution of Genetic Variation

Based on the geographical patterns of plastid and ribosomal sequence variation, three main evolutionary entities can be discerned in the *C. albidus*–*C. creticus* complex:

- Western Mediterranean distributed *C. albidus* that appeared genetically homogeneous and was typically characterized by *trnL-trnF* chlorotype A and ribotype B (or its rarer variants).
- Eastern Mediterranean distributed *C. creticus*, below referred to as Eastern Mediterranean clade (EM clade), that was genetically homogeneous and exhibited *trnL-trnF* chlorotype A (or its rarer variants) and ribotype A (or its rarer variants).
- Western and Central Mediterranean distributed *C. creticus*, below referred to as Western Mediterranean clade (WM clade), that appeared conspicuously heterogeneous and was characterized by *trnL-trnF* chlorotype B, C, or E and dissimilar co-occurring, putative paralogues ribotypes (typically ribotype A together with one regionally distributed ribotype). Based on the regionally distributed ITS paralogs, two large sub-entities within the WM clade became obvious. One including the populations from Spain, Italy, and Croatia (predominantly characterized by ribotype D) and the second one including populations from Albania and Greece (predominantly characterized by ribotype C).

The four species of subgenus *Cistus* were described to freely hybridize with each other [7,17,33]. The here observed distribution patterns of chloro- and ribotype variants indicated an intertaxon genetic exchange in two geographical regions. The presence of ribotype A in two native populations of France and an Italian *C. albidus* Seedbank accession might indicate gene flow from *C. creticus* into *C. albidus*. The distribution of *C. albidus* and *C. creticus* was described to overlap in this geographical region [11]. However, during our plant collection along the Ligurian and Tyrrhenian Sea, no sites with close co-occurring *C. creticus* and *C. albidus* were located. The presence of a *C. creticus* specific ribotype in *C. albidus* could therewith also be a relic of a common ancestor species that was preserved in the easternmost populations of *C. albidus*. In parts of mainland Greece and the Peloponnesus there was evidence for genetic exchange between the WM and EM clade of *C. creticus*. Populations from the provinces Central and East Macedonia in Greece exhibited *trnL-trnF* chlorotype A that was attributed to the EM clade together with diverse rare variants of ribotype C, characteristic for the WM clade. Additionally, the elevated number of individual ITS sequence characteristics and rare ribotypes in Greek populations might be indicative of a higher genetic diversity when the characteristics of the two lineages run into each other.

### 4.3. A Hypothesis about Evolutionary Relationships in the C. albidus-C. creticus Complex and Biogeographic Considerations

Molecular clock calculations have dated the last common ancestor of the closely related *C. albidus, C. creticus* and *C. heterophyllus* with 0.19 (0.33–0.01) Myr to the Chibanian age. The diversification point of *C. albidus* and the WM clade of *C. creticus* (the in Albania/Greece distributed sub-entity) was dated to 0.04 (0.13–0.002) Myr [10] and therewith as a rather recent event that, when emanating from the proposed 0.04 Myr, for example, would coincide with the arrival of the anatomically modern humans in Europe.

Based on earlier hypotheses [8,10,11] and the distribution of genetic variation observed here, we act on the assumption of a primordial, widely distributed *Cistus* species whose continuous distribution in Europe has split sometime during the Pleistocene. *Cistus albidus* and Eastern Mediterranean *C. creticus* sharing chlorotype A might represent the progeny of peripheral relict populations [44,45], with *C. albidus* as a direct descendant in the West and the EM clade of *C. creticus* as the corresponding counterpart in the East. In the case of Eastern Mediterranean *C. creticus*, this hypothesis seems to be confirmed by its genetic homogeneity and its disjunct distribution apart from its closest genetic relative and the diversity centre of the genus. Due to reproductive isolation (beside *C. creticus*, solely three *Cistus* species from the white- and whitish-pink-flowered *Cistus* clade are native to Turkey and the Near East) and short diversification time, Eastern Mediterranean *C. creticus* could have kept its original characteristics.

In the Western Mediterranean, the situation appears to be more complex. Spain and Morocco with twelve *Cistus* species each, constitute the actual diversity centre of the genus. Four of the nine purple-flowered *Cistus* species, the four members of subgenus *Cistus*, are native to the Iberian Peninsula and Morocco. Among them, *C. crispus* (distributed from North Africa to Italy) is distinct whereas *C. albidus* and the in Spain exceedingly rare *C. creticus* and *C. heterophyllus* (distributed in North Africa and rarely in Spain) appear to be nearer associated [11]. The close relationship of the latter three species can conclusively be explained by recent or rapid diversification from a common ancestor but might furthermore be rooted in reticulation/hybridization. European *C. albidus* appeared genetically homogeneous and exhibited no clear evidence for a hybridogeneous speciation. In *C.* × *clausonii* nothosubsp. *crespoi* P.P. Ferrer and E. Laguna, the one hybrid of *C. heterophyllus* × *C. albidus* native in Spain*,* chloroplast heteroplasmy was revealed that was hypothesized to indicate ancient hybridization with an unidentified *Cistus* taxon [33]. Within the WM clade of *C. creticus* the simultaneous occurrence of distantly related ribotypes and the interspecifically shared *trnL-trnF* chlorotype C strongly indicate hybridogeneous (reticulate) speciation. From the observed genetic characteristics, a specific contribution of potentially involved taxa could not clearly be identified. The in *C. creticus* continuously distributed ribotype A was attributed to an ancient widespread ancestor species or immediate descendants thereof, based on the dominant appearance of this ribotype in the EM clade. The regionally co-occurring ITS paralogs appear to be a younger characteristic that seems to have derived from the main ribotype of *C. albidus*. The chlorotypes of Spanish and Central Mediterranean *C. creticus* are (according to Genbank sequences) either shared with *C. albidus* and *C. heterophyllus* or are strongly associated with further chlorotypes of *C. heterophyllus*. Beside the cryptic combination of genetic characteristics, Western Mediterranean *C. creticus* exhibits furthermore a conspicuous phytochemical specialty. The species of subgenus *Cistus* are usually characterized by flavonols [24,25]. Aside from these character compounds, populations of the WM clade, especially the one sub-entity present in Spain, Italy and Croatia, exhibit higher amounts of punicalagin derivatives [25], a chemical characteristic that is unique in this form in subgenus *Cistus* and is usually associated with white-flowered *Cistus* species [24,25,46]. The spectrum of non-volatile compounds of *Cistus* species was suspected to be strongly related to evolutionary events [24,25]. The specific phytochemical characteristic of Western Mediterranean *C. creticus* could constitute the preservation of a very ancient, primordial feature or could indicate hybridization and a therewith (re-)acquired phytochemical trait. The regionally distributed ribotypes of Western Mediterranean *C. creticus* seem to have also preserved ancient or acquired nucleotide polymorphisms. Ribotypes C and D are characterized by nucleotide polymorphisms that are not present in *C. albidus* and Eastern Mediterranean *C. creticus* but are shared with *C. heterophyllus* and white-flowered *C. salviifolius* (ribotype C) or *C. monspeliensis* (ribotype D). Interestingly, also in the EM clade, a distinctive phytochemical trait was supplemented by specific local ITS characteristics. Cypriot *C. creticus* exhibited also high amounts of punicalagin derivatives whereas these compounds seem to be of marginal importance in populations from Eastern Mediterranean mainland localities [25]. Two of the Cypriot accessions exhibited nucleotide polymorphisms that were not present in any other *Cistus* sample or were shared with *C. crispus*, *C. heterophyllus* and the white-flowered species. It is conceivable that populations on the biogeographically isolated island of Cyprus have rather preserved ancient traits than continental mainland populations. Cypriot populations might be a rewarding object of study for deeper insights into the evolutionary processes within subgenus *Cistus*.

The geographical origin of the WM clade was located to the Iberian Peninsula as this is the region where *C. creticus* and its possible progenitors or hybridization partners *C. albidus* and *C. heterophyllus* occur geographically close. Support comes also from [8] and [10] who concluded that dispersal and colonization of *Cistus* species across the Mediterranean basin has taken place after divergence and species formation in its West. When regarding chloroplast data, especially the slightly more informative *trnL-trnF* data, these hypotheses seem to apply to the observed distribution patterns. The migration movement of the WM clade of *C. creticus* appears to be a continuous one, starting with the basic chlorotype C in Spain and continuing eastwards with chlorotype D to Corsica/Sardinia, Italy, Albania and Greece and chlorotype E from Italy to Croatia. In sharp contrast to this, the ribotype distribution in Western Mediterranean *C. creticus* is evidently a discontinuous one. The strictly regionally distributed ribotypes C and D share none of their characteristic variable nucleotide positions and both ribotypes appear to have originated separately from the basic ribotype of *C. albidus.* The decreasing relevance of punicalagin derivatives in *C. creticus* populations eastwards of Albania [25], underlines the genetic divergence between the two sub-entities of the WM clade. Based on these findings, it could be hypothesized that formation or range expansion of the Western Mediterranean clade was a repeated event. Ribotype C and ribotype D may represent consecutive migration waves out of refugia on the Iberian Peninsula that followed consecutive cooling periods or are coincided with massive regional ecological changes as e.g., could have been caused by a super-eruption at the Phlegrean fields that buried wide areas of South-East Europe under massive volcanic ash deposits about 0.04 Ma ago [47,48]. Alternatively, ribotype C and ribotype D might represent the progeny of geographically distinct relict populations that have re-expanded from different south-European refugia (Sicily, Peloponnese). Very recently then, increasing anthropogenic influence on ecosystems of coastal near areas might have established favourable conditions for a further range expansion. On the Istrian peninsula (Croatia), human impact on coastal ecosystems began about 5000 years ago and was described as one of the main drivers of a long-term ecological change that led to the replacement of coastal-near woods by ‘shrubland’ dominated, amongst others, by *Cistus* [49].

Three populations of *C. creticus* unexpectedly exhibited genetic characteristics that did not fit well in the overall distribution patterns of chloro- and ribotypes. Plants raised from seeds that originated from the Crimean Peninsula and Cyprus, as well as one Turkish accession, exhibited ribotype D (ribotype information for the one Turkish accession is missing) and *trnL-trnF* chlorotype D, a combination of characteristics that was, with one exception detected in Croatia, strongly restricted to Italy. Ancient Greek settlements are well documented for both areas, the Crimean Peninsula and Cyprus. The presence of Mediterranean Flora on the Crimean Peninsula appears to be the result of a complex process involving biogeographic processes and human-aided introduction of species [50]. When considering the disjunct presence of genetic characteristics, it can be hypothesized that emigrants from the historical Magna Graecia, actively or passively, brought along plant material or seeds from the South-Italian coast.

## 5. Concluding Remarks

*Cistus albidus* and *C. creticus* can be clearly differentiated and identified when combining sequence information from nuclear ITS and chloroplast *trnL-trnF* and considering the intraspecific genetic diversity of *C. creticus*. Genetically, C. *creticus* was confirmed as a heterogeneous species that integrates two major evolutionary lineages with clearly different genetic characteristics. The EM clade of *C. creticus* seems to represent old and ancestral characteristics. The genetically more complex WM clade seems to be the result of hybridization and recurrent or parallel migration movements. The genetic disparity among EM and WM clade appears to be reflected by differences in the non-volatile compound composition but could not clearly be associated with consistent macromorphological tendencies towards one of the two lineages. Traits such as the absence/presence or density of non-glandular and glandular trichomes or leaf characteristics varied highly within and between natural populations of Spain, Italy, Croatia, Albania, and Cyprus. This high morphological diversity is not surprising when considering the high level of genetic diversity within the WM clade and the indicated complex evolutionary history, but this impedes the identification of relevant discriminating characteristics. Potentially, thorough multivariate analysis of trait variation can aid identification of relevant distinctive characters. Based on the nuclear and the two chloroplast markers used, besides the inter-regional disparity of genetic characteristics, within *C. creticus,* there was no evident correlation between DNA characteristics and the designation of plant material to varieties or subspecies based on morphological characteristics. These results are in accordance with a previous investigation that related small scale morphological diversity more to local ecological factors (as e.g., bedrock) than to characteristics of chloroplast markers [16]. Based on these results, one could debate whether the currently often applied species concept of *C. creticus* with its three recognized subspecies *creticus*, *corsicus* and *eriocephalus* [13] must be better reworked. Our results argue for the recognition of two (genetically but not morphologically distinguishable) subspecies within *C. creticus* corresponding to the two major evolutionary lineages, here referred to as WM clade (*C. creticus* subsp. *hesperius*) and EM clade (*C. creticus* subsp. *orientalis*). As some Greek accessions with combined genetic characteristics indicated genetic exchange, this subdivision into two subspecies seems more adequate than a two-species concept. The three morphological and (regarding volatile content and composition) chemotypic variants presently treated as subspecies are hard to grasp. [15] were able to differentiate between *C. creticus* ssp*. corsicus* and subsp. *eriocephalus* from Corsica/Sardinia based on volatiles and ISSR markers, but their results are difficult to integrate as accessions from these islands appear as very distinct group in our chlorotype networks. As both densely glandular and viscid plants (presently recognized as subsp. *corsicus* and subsp. *creticus*) and much less or not glandular but conspicuously pubescent plants (presently recognized as subsp. *eriocephalus*) were described within both evolutionary lineages, we would propose to downgrade their taxonomic status from subspecies to form level.

## Figures and Tables

**Table 1:** Variable alignment positions and chlorotype (CT) designation in the *trnL-trnF* intergenic spacer of *C. creticus* (cre), *C. albidus* (alb) and *C. × canescens* (can). Only chlorotypes from plants collected during this investigation were included. Alignment position and consensus sequence are indicated in the header. The two, small poly-T stretch attached indels at alignment positions 261/262 were present in 71/2 individuals of the respective chlorotypes (C, D and E).

			0	0	0	0	1	1	2	2	2	2	2	2	3	3	3
			4	5	8	9	6	6	6	6	9	9	9	9	5	5	6
			8	0	6	1	0	6	1	2	3	4	5	6	7	9	6
**CT**	**Species**	**n**	**C**	**T**	**T**	**G**	**T**	**C**	**-**	**-**	**-**	**-**	**-**	**-**	**T**	**C**	**T**
A	cre/alb/can	55/30/1	.	G	.	T	G	.	.	.	.	.	.	.	G	.	.
B	cre	1	.	G	G	T	G	.	.	.	.	.	.	.	G	.	.
C	cre/can	6/1	T	.	.	.	.	.	T	.	.	.	.	.	.	.	.
D	cre	48	T	.	.	.	.	.	T	T	C	T	T	T	.	T	-
E	cre	16	T	.	.	.	.	T	T	.	C	T	T	T	.	T	-

**Table 2:** Variable sites and chlorotype (CT) designation in the *rpl32-trnL* intergenic spacer of *C. creticus* (cre), *C. albidus* (alb) and *C.* × *canescens* (can). Only chlorotypes of plants collected during this investigation were included. Alignment positions and consensus sequence are indicated in the header.

			1	3	4	4	7
			9	1	1	3	3
			4	5	1	5	9
**CT**	**Species**	**n**	**G**	**C**	**G**	**A**	**G**
A	cre/alb/can	51/26/1	.	.	.	.	.
B	cre	3	.	A	.	.	.
C	cre	1	.	.	.	.	T
D	cre/can	59/1	T	.	T	G	.

**Table 3:** Variable sites and ribotype (RT) designation in the ITS alignment of *C. creticus* (cre) and *C. albidus* (alb). Solely sequence ribotypes detected during this investigation are included. Alignment position and consensus sequence is indicated in the header. Asterisks refer to ribotype sequences extracted solely from heterozygous plants. ● = Insertion (CGTCCT, alignment position bp 569 to bp 574) that discriminates ribotype A (and its variants) from all further ribotypes.

			0	0	1	1	1	1	1	1	1	2	2	3	4	4	4	4	4	5	5	5	5	6	6	6	6	6
			1	6	0	1	5	5	5	5	8	2	4	8	4	5	5	5	7	2	6	9	9	0	3	4	4	5
			7	6	0	1	0	1	2	3	8	9	1	6	2	3	4	5	5	6	9	1	8	6	2	0	7	1
**RT**	**Species**	**n**	**C**	**G**	**C**	**C**	**G**	**T**	**C**	**G**	**G**	**C**	**C**	**C**	**G**	**C**	**T**	**G**	**A**	**C**	**●**	**G**	**A**	**G**	**C**	**C**	**C**	**C**
A	cre/alb	48/3	.	.	.	.	.	.	.	.	.	.	.	.	.	.	.	.	.	.	-	.	.	.	T	.	.	.
A1	cre	2	.	.	.	.	.	.	.	.	.	.	.	.	.	.	.	.	.	.	-	A	.	.	T	.	.	.
A2	cre	1	.	S	.	.	.	.	.	.	.	.	.	.	.	.	.	.	.	.	-	.	.	.	T	.	.	.
A3	cre	1 *	.	.	.	T	.	.	.	.	.	.	.	T	.	.	.	.	.	.	-	.	.	.	T	.	.	.
A4	cre	1 *	.	.	.	.	T	.	.	.	.	.	.	.	.	.	.	.	.	.	-	.	.	.	T	.	.	.
A5	cre	1	.	.	.	.	.	.	.	.	.	.	.	.	R	.	.	.	.	.	-	.	.	.	T	.	.	.
A6	cre	1 *	.	.	.	.	.	.	.	.	.	.	.	.	.	.	.	.	R	.	-	.	.	.	T	.	.	.
A7	cre	1 *	.	.	.	.	.	.	.	.	.	.	.	.	.	.	.	.	.	Y	-	.	.	.	T	.	.	.
A8	cre	1 *	.	.	.	.	.	.	.	.	.	.	.	.	.	.	.	.	.	.	-	.	.	.	T	.	.	G
A9	cre	1 *	.	.	.	.	.	.	.	.	.	.	.	.	.	.	.	.	.	.	-	.	.	.	T	.	G	G
B	alb	13	.	.	.	.	.	.	.	.	.	.	.	.	.	.	.	.	.	.	●	.	.	.	.	.	.	.
B1	alb	2	.	.	.	.	.	Y	.	.	.	.	.	.	.	.	.	.	.	.	●	.	.	.	.	.	.	.
B2	alb	7	.	.	.	.	.	C	.	.	.	.	.	.	.	.	.	.	.	.	●	.	.	.	.	.	.	.
C	cre	18	.	.	.	.	.	.	.	T	.	.	.	.	.	.	C	A	.	.	●	.	.	.	.	.	.	.
C1	cre	1	S	.	.	.	.	.	.	T	.	.	.	.	.	.	C	A	.	.	●	.	.	.	.	.	.	.
C2	cre	1	S	.	.	.	.	.	.	T	.	.	M	.	.	.	C	A	.	.	●	.	.	.	.	.	.	.
C3	cre	1	.	.	.	.	.	.	.	T	.	Y	.	.	.	.	C	A	.	.	●	.	.	.	.	.	.	.
C4	cre	1	.	.	.	.	.	.	.	T	A	.	.	.	.	.	C	A	.	.	●	.	.	.	.	.	.	.
C5	cre	1	.	.	.	.	.	.	.	T	.	.	.	.	.	T	C	A	.	.	●	.	.	.	.	.	.	.
C7	cre	1 *	.	.	.	.	.	.	.	T	.	.	.	.	.	.	.	A	.	.	●	.	.	.	.	.	.	.
C8	cre	1	.	.	.	.	.	.	S	T	.	.	.	.	.	.	C	A	.	.	●	.	.	.	.	.	.	.
C10	cre	1	.	.	Y	.	.	.	.	K	.	.	.	.	.	.	Y	A	.	.	●	.	R	.	Y	.	.	.
D	cre	27	.	.	.	.	.	.	.	.	.	.	.	.	.	.	.	.	G	.	●	.	.	.	.	.	.	.
D1	cre	1	.	.	.	.	.	.	.	.	.	.	.	.	.	.	.	.	G	T	●	.	.	.	.	.	.	.
D2	cre	1	.	.	.	.	.	.	.	.	.	.	.	.	.	.	.	.	G	Y	●	.	.	.	.	.	.	.
D3	cre	1 *	.	.	.	.	.	.	.	.	.	.	.	.	.	.	.	.	R	.	●	.	R	.	Y	.	.	.
E	cre	1 *	.	.	.	.	.	.	.	.	.	.	.	.	.	.	.	.	.	.	●	.	.	.	.	A	.	.
E1	cre	1 *	.	.	.	.	T	.	.	.	.	.	.	.	.	.	.	.	.	.	●	.	.	T	.	A	.	.
F	cre	2 *	.	.	T	T	.	.	.	.	.	.	.	.	.	.	.	R	.	.	●	.	G	A	T	.	.	.

## Data Availability

Data is contained within the article.

## Supplementary Materials

The following are available online at https://www.mdpi.com/article/10.3390/plants10081619/s1, Table S1: List of plant material sampled and sequenced for this investigation: Geographical origin of wild populations and Seed bank accession numbers; NCBI accession numbers of ITS ribotypes, *trnL-trnF* chlorotypes and *rpl32-trnL* chlorotypes. Designation of plant material from wild populations was performed based on local floras (references are provided as footnotes). Table S2: Variable alignment positions and chlorotypes of the complete *trnL-trnF* alignment (including sequence information of this investigation, available GenBank accessions of species of the purple-flowered *Cistus* clade and four additional white- or pink-flowered *Cistus* species). Table S3: Variable sites and chlorotypes of the extended *rpl32-trnL* alignment (combining *rpl32-trnL* sequence information of this investigation, available GenBank accessions of species of the purple-flowered *Cistus* clade and four additional white- or pink-flowered *Cistus* species). Table S4: Variable alignment positions and ribotypes of the complete ITS sequence alignment (combining ITS sequence information of this investigation, available GenBank accessions of species of the purple-flowered *Cistus* clade and four additional white- or pink-flowered *Cistus* species).

## References

[1] Dunal M.F., De Candolle A.P. (1824). Cistineae. Prodromus Systematis Naturalis Regni Vegetabilis I.

[2] Spach E. (1836). Conspectus monographiae Cistacearum. Ann. Sci. Nat. Bot..

[3] Willkomm M. (1856). Icones et Descriptiones Plantarum Novarum Criticarum et Rariorum Europae Austro-Occidentalis Praecipue Hispaniae torn. 2.

[4] Grosser W., Engler A. (1903). Cistaceae. Das Pflanzenreich.

[5] Dansereau P.M. (1939). Monographie du genre *Cistus*. Boissiera.

[6] von Raab-Straube E. *Cistaceae*. Euro+Med Plantbase—The Information Resource for Euro-Mediterranean Plant Diversity. http://ww2.bgbm.org/EuroPlusMed/.

[7] Demoly J.P., Montserrat P., Castroviejo S. (1993). Cistus. Flora Ibérica.

[8] Guzmán B., Vargas P. (2005). Systematics, character evolution, and biogeography of *Cistus* L. (Cistaceae) based on ITS, *trnL-trnF*, and matK sequences. Mol. Phylogenet. Evol..

[9] Guzmán B., Vargas P. (2009). Historical biogeography and character evolution of Cistaceae (Malvales) based on analysis of plastid rbcL and *trnL-trnF* sequences. Org. Divers. Evol..

[10] Guzmánn B., Lledó M.D., Vargas P. (2009). Adaptive radiation in Mediterranean *Cistus* (Cistaceae). PLoS ONE.

[11] Civeyrel L., Leclercq J., Demoly J.-P., Agnan Y., Quèbre N., Pélissier C., Otto T. (2011). Molecular systematics, character evolution, and pollen morphology of *Cistus* and *Halimium* (Cistaceae). Plant Syst. Evol..

[12] Greuter W., Raus T. (1981). Med-Checklist Notulae, 4. Willdenowia.

[13] Greuter W., Burdet H.M., Long G. Med Checklist. A Critical Inventory of Vascular Plants of the Circum-Mediterranean Countries. http://ww2.bgbm.org/mcl/home.asp.

[14] Demetzos C., Anastasaki T., Perdetzoglou D. (2002). A chemometric interpopulation study of the essential oils of *Cistus creticus* L. growing in Crete (Greece). Zeitschrift Nat. C.

[15] Paolini J., Falchi A., Quilichini Y., Desjobert J.-M., De Cian M.-C., Varesi L., Costa J. (2009). Morphological, chemical and genetic differentiation of two subspecies of *Cistus creticus* L. (*C. creticus* subsp. eriocephalus and C. creticus subsp. corsicus). Phytochemistry.

[16] Falchi A., Paolini J., Desjobert J.-M., Melis A., Costa B., Varesi L. (2009). Phylogeography of *Cistus creticus* L. on Corsica and Sardinia inferred by the TRNL-F and RPL32-TRNL sequences of cpDNA. Mol. Phylogenet. Evol..

[17] Gómez Navarro J., Roselló Gimeno R. (2008). *Cistus* × *canescens* Sweet, estepa silvestre en la Península Ibérica. Sabuco Rev. Estud. Albacet..

[18] Gómez Navarro J., Peris Gisbert J.B., Valdés Franzi A., Sanchis Duato E., Roselló Gimeno R. (2011). Plantas de interes del NE de la provincia de Albacete e inmediaciones de la provincia de Valencia, VI. Sabuco Rev. Estud. Albacet..

[19] Dimas K., Demetzos C., Vaos V., Ioannidis P., Trangas T. (2001). Labdane type diterpenes down-regulate the expression of c-Myc protein, but not of Bcl-2, in human leukemia T-cells undergoing apoptosis. Leuk. Res..

[20] Dimas K., Papadaki M., Tsimplouli C., Hatziantoniou S., Alevizopoulos K., Pantazis P., Demetzos C. (2006). Labd-14-ene-8,13-diol (sclareol) induces cell cycle arrest and apoptosis in human breast cancer cells and enhances the activity of anticancer drugs. Biomed. Pharmacother..

[21] Matsingou C., Dimas K., Demetzos C. (2006). Design and development of liposomes incorporating a bioactive labdane-type diterpene: In vitro growth inhibiting and cytotoxic activity against human cancer cell lines. Biomed. Pharmacother..

[22] Papaefthimiou D., Papanikolaou A., Falara V., Givanoudi S., Kostas S., Kanellis A.K. (2014). Genus *Cistus*: A model for exploring labdane-type diterpenes’ biosynthesis and a natural source of high value products with biological, aromatic, and pharmacological properties. Front. Chem..

[23] Skorić M., Todorović S., Gligorijević N., Janković R., Zivković S., Risti M., Radulovi M. (2012). Cytotoxic activity of ethanol extracts of in vitro grown *Cistus creticus* subsp. creticus L. on human cancer cell lines. Ind. Crop. Prod..

[24] Barrajón-Catalán E., Fernández-Arroyo S., Roldán C., Guillén E., Saura D., Segura-Carretero A., Micol V. (2011). A systematic study of the polyphenolic composition of aqueous extracts deriving from several *Cistus* genus species: Evolutionary relationships. Phytochem. Anal..

[25] Lukas B., Bragagna L., Starzyk K., Labedz K., Stolze K., Novak J. (2021). Polyphenol Diversity and Antioxidant Activity of European *Cistus creticus* L. (Cistaceae) Compared to Six Further, Partly Sympatric *Cistus* Species. Plants.

[26] Doyle J.J., Doyle J.L. (1990). Isolation of Plant DNA from Fresh Tissue. Focus.

[27] Schmiderer C., Lukas B., Novak J. (2013). Effect of different DNA extraction methods and DNA dilutions on the amplification success in the PCR of different medicinal and aromatic plants. Zeitschrift Arznei Gewürzpflanzen.

[28] White T.J., Bruns T.D., Lee S.B., Taylor J.W., Innis M.A., Gelfand D.H., Sninsky J.J., White T.J. (1990). Amplification and direct sequencing of fungal ribosomal RNA Genes for phylogenetics. PCR Protocols, a Guide to Methods and Applications.

[29] Downie S.R., Katz-Downie D.S. (1996). A molecular phylogeny of Apiaceae subfamily Apioideae: Evidence from nuclear ribosomal DNA internal transcribed spacer sequences. Am. J. Bot..

[30] Taberlet P., Gielly L., Pautou G., Bouvet J. (1991). Universal primers for amplification of three non-coding regions of chloroplast DNA. Plant Mol. Biol..

[31] Shaw J., Lickey E.B., Schilling E.E., Small R. (2007). Comparison of whole chloroplast genome sequences to choose noncoding regions for phylogenetic studies in angiosperms: The tortoise and the hare III. Am. J. Bot..

[32] Clark A.G. (1990). Inference of haplotypes from PCR-amplified samples of diploid populations. Mol. Biol. Evol..

[33] Pawluczyk M., Weiss J., Vicente-Colomer M.J., Egea-Cortines M. (2012). Two alleles of rpoB and rpoC1 distinguish an endemic European population from *Cistus heterophyllus* and its putative hybrid (*C*. × *clausonis*) with *C. albidus*. Plant Syst. Evol..

[34] Aparicio A., Martín-Hernanz S., Parejo-Farnés C., Arroyo J., Lavergne S., Yeşilyurt E.B., Zhang M.-L., Rubio E., Albaladejo R.G. (2017). Phylogenetic reconstruction of the genus *Helianthemum* (Cistaceae) using plastid and nuclear DNA-sequences: Systematic and evolutionary inferences. Taxon.

[35] R Core Team R: A Language and Environment for Statistical Computing.

[36] Paradis E. (2010). Pegas: An R package for population genetics with an integrated–modular approach. Bioinformatics.

[37] Jombart T. (2008). adegenet: A R package for the multivariate analysis of genetic markers. Bioinformatics.

[38] Jombart T., Ahmed I. (2011). adegenet 1.3-1: New tools for the analysis of genome-wide SNP data. Bioinformatics.

[39] Kamvar Z.N., Tabima J.F., Grünwald N.J. (2014). Poppr: An R package for genetic analysis of populations with clonal, partially clonal, and/or sexual reproduction. PeerJ.

[40] Kamvar Z.N., Brooks J.C., Grünwald N.J. (2015). Novel R tools for analysis of genome-wide population genetic data with emphasis on clonality. Front. Genet..

[41] Bache S.M., Wickham H. (2014). magrittr: A Forward-Pipe Operator for R. R Package Version 1.5. https://CRAN.R-project.org/package=magrittr.

[42] Zhang D., Sang T. (1999). Physical mapping of ribosomal RNA genes in peonies (*Paeonia*, *Paeoniaceae*) by fluorescent in situ hybridization: Implications for phylogeny and concerted evolution. Am. J. Bot..

[43] Totta C., Rosato M., Ferrer-Gallego P., Luccese F., Rossello J.A. (2016). Temporal frames of 45S rDNA site-number variation in diploid plant lineages: Lessons from the rock rose genus *Cistus* (*Cistaceae*). Biol. J. Linn. Soc..

[44] Hewitt G. (2000). The genetic legacy of the Quaternary ice ages. Nature.

[45] Medail F., Diadema K. (2009). Glacial refugia influence plant diversity patterns in the Mediterranean Basin. J. Biogeogr..

[46] Aničić N., Patelou E., Papanikolaou A., Kanioura A., Valdesturli C., Arapitsas P., Skoric M., Dragicevic M., Gasic U., Koukounaras A. (2021). Comparative metabolite and gene expression analyses in combination with gene characterization revealed the patterns of flavonoid accumulation during *Cistus creticus* subsp. creticus fruit development. Front. Plant Sci..

[47] Fedele F.G., Giaccio B., Isaia R., Orsi G. (2003). The Campanian Ignimbrite Eruption, Heinrich Event 4, and Palaeolithic Change in Europe: A High-Resolution Investigation. Volcanism Earth’s Atmos. Geophys. Monogr. Ser..

[48] Costa A., Folch A., Macedonio G., Giaccio B., Isaia R., Smith V.C. (2012). Quantifying volcanic ash dispersal and impact of the Campanian Ignimbrite super-eruption. Geophys. Res. Lett..

[49] Kaniewski D., Marriner N., Morhange C., Rius D., Carre M.-B., Faivre S., Van Campo E. (2018). Croatia’s mid-Late Holocene (5200-3200 BP) coastal vegetation shaped by human societies. Quat. Sci. Rev..

[50] Cordova C. (2016). The Mediterraneanization of Crimea: Physical and cultural processes in landscape transformation. Méditerranée.

